# Platoon Interactions and Real-World Traffic Simulation and Validation Based on the LWR-IM

**DOI:** 10.1371/journal.pone.0144798

**Published:** 2016-01-05

**Authors:** Kok Mun Ng, Mamun Bin Ibne Reaz

**Affiliations:** 1 Faculty of Electrical Engineering, Universiti Teknologi Mara, Shah Alam, Selangor, Malaysia; 2 Department of Electrical, Electronic and Systems Engineering, Faculty of Engineering and Built Environment, Universiti Kebangsaan Malaysia, UKM Bangi, Selangor, Malaysia; Beihang University, CHINA

## Abstract

Platoon based traffic flow models form the underlying theoretical framework in traffic simulation tools. They are essentially important in facilitating efficient performance calculation and evaluation in urban traffic networks. For this purpose, a new platoon-based macroscopic model called the LWR-IM has been developed in [1]. Preliminary analytical validation conducted previously has proven the feasibility of the model. In this paper, the LWR-IM is further enhanced with algorithms that describe platoon interactions in urban arterials. The LWR-IM and the proposed platoon interaction algorithms are implemented in the real-world class I and class II urban arterials. Another purpose of the work is to perform quantitative validation to investigate the validity and ability of the LWR-IM and its underlying algorithms to describe platoon interactions and simulate performance indices that closely resemble the real traffic situations. The quantitative validation of the LWR-IM is achieved by performing a two-sampled t-test on queues simulated by the LWR-IM and real queues observed at these real-world locations. The results reveal insignificant differences of simulated queues with real queues where the p-values produced concluded that the null hypothesis cannot be rejected. Thus, the quantitative validation further proved the validity of the LWR-IM and the embedded platoon interactions algorithm for the intended purpose.

## Introduction

Traffic flow models are underlying “engines” of traffic simulation tools. Various traffic models that emulate the behavior of the urban traffic system over time and space to predict traffic states have been developed. These models include microscopic and macroscopic traffic flow models. Microscopic models capture the behavior of every single vehicle such as driver parameters (e.g. aggressiveness, reaction time, etc.) and parameters of each vehicle (e.g. mass, acceleration, etc.) moving in the traffic stream. Hence, the behavior of all the individual vehicles and their interactions can be simulated. Existing models that describe the traffic situation microscopically include the car following model [2–7] and the cellular automaton [8]. The state of the traffic system such as link section speed, flow, travel time, delay, stop time, intersection turnings and etc. can be predicted from these models via simulation. On the other hand, macroscopic models do not describe the traffic situation at the level of independent vehicles. Variables such as traffic flow, density and average velocity are used to provide aggregated information of multiple vehicles. The macroscopic model typically defines the relationship between traffic density, traffic flow and traffic velocity [9–10]. Besides, there are macroscopic models that were derived from microscopic flow models by converting the single entity characteristics to system level aggregated characteristics [11–12].

One of the inherent researches on traffic flow models involves the progression of platoons in coordinated signalized urban networks. Hence, macroscopic traffic flow models were developed to describe the platoon progression in these urban networks. These platoon progression models that enable performance evaluation of signalized urban networks can be attributed to the platoon dispersion model (PDM) [13], the cell transmission model (CTM) [14], Shockwave tracking model [15] and the Highway Capacity Manual 2000 (HCM 2000) [16]. Development of platoon-based traffic flow model is still ongoing with the purpose to enhance and improve current existing models [17–21].

In a previous work, the authors proposed a new platoon traffic flow model to address limitations in some of the current traffic flow models [1]. The model which is renamed as LWR-IM in this paper is based on the integration of Lighthill-Witham-Richard (LWR) model [9–10] and the Rakha vehicle dynamics model [22]. In [1], the authors adapted continuous timed Petri nets with variable speeds (VCPN) from Tolba et al. [23, 24] to describe the LWR through a segmentation approach of the urban arterial or link. The LWR-IM is based on the notion that speeds in each segments of the link could be estimated by the Rakha model [22]. It could describe efficiently both under-saturated and oversaturated traffics and does not require additional mathematical expressions to address oversaturation such as in the PDM. The LWR-IM is also less computationally compared to CTM due to the lesser number of discrete variables handled. Finally, the notion to analyse performance using input-output method simplifies analysis provided previously by Shockwave tracking model. A comparative assessment was conducted using a simple case study. Analytical validation of results simulated by our model in [1] with CTM, PDM and HCM 2000 reveals that the model is valid and feasible.

Our work in [1] successfully described dynamics of a single platoon moving along a 700m arterial. However, in actual situations, an urban arterial particularly class I arterial may receive a few platoons from its upstream signal phases. The movement and interactions of two or more of these platoons moving along the arterial should be appropriately described. Hence, in this paper, we aim to further enhance the LWR-IM with algorithms to describe the interactions of platoons moving in real-world urban arterial. Upon enhancement of the LWR-IM with the proposed algorithms, the research also aims to further quantitatively validate the enhanced LWR-IM with real data collected from real-world traffic. This method of comparing simulated results with real data aims to investigate the ability of the model to simulate outputs that resembles actual situations can also be referred in [25–28]. Hence, the LWR-IM is applied to a class I arterial located in the city of Shah Alam, Malaysia to simulate interaction of two moving platoons. The LWR-IM is also applied to a class II urban arterial located in the city of Klang, Malaysia for the same purpose. Performances indices such as residue queue Q_r_ and queue at begin of green Q_s_ simulated by the LWR-IM are compared to real queues observed at both locations to ascertain the feasibility and accuracy of the LWR-IM. The comparison is conducted using a two-sample t-test [29] to evaluate whether the simulated and real queues are significantly different from each other. If the t-test reveals insignificant differences, the model is deemed valid and capable to describe the real-world situations. The feasibility of the model is also further evaluated with statistical measures, i.e. the mean absolute error.

The subsequent section presents concisely the LWR-IM model published by the authors in [1]. Section 3 shows the proposed algorithm that implements platoon interactions in the LWR-IM. Section 4 details the experimental and simulation procedures in applying and implementing the LWR-IM into the class I and class II arterials. In addition, the validation and analysis procedures are explained in section 4. Section 5 provides validation results on the queues simulated with real queues observed at both locations. Concluding remarks are given in section 6.

## The LWR-IM Model

**Fig 1:**
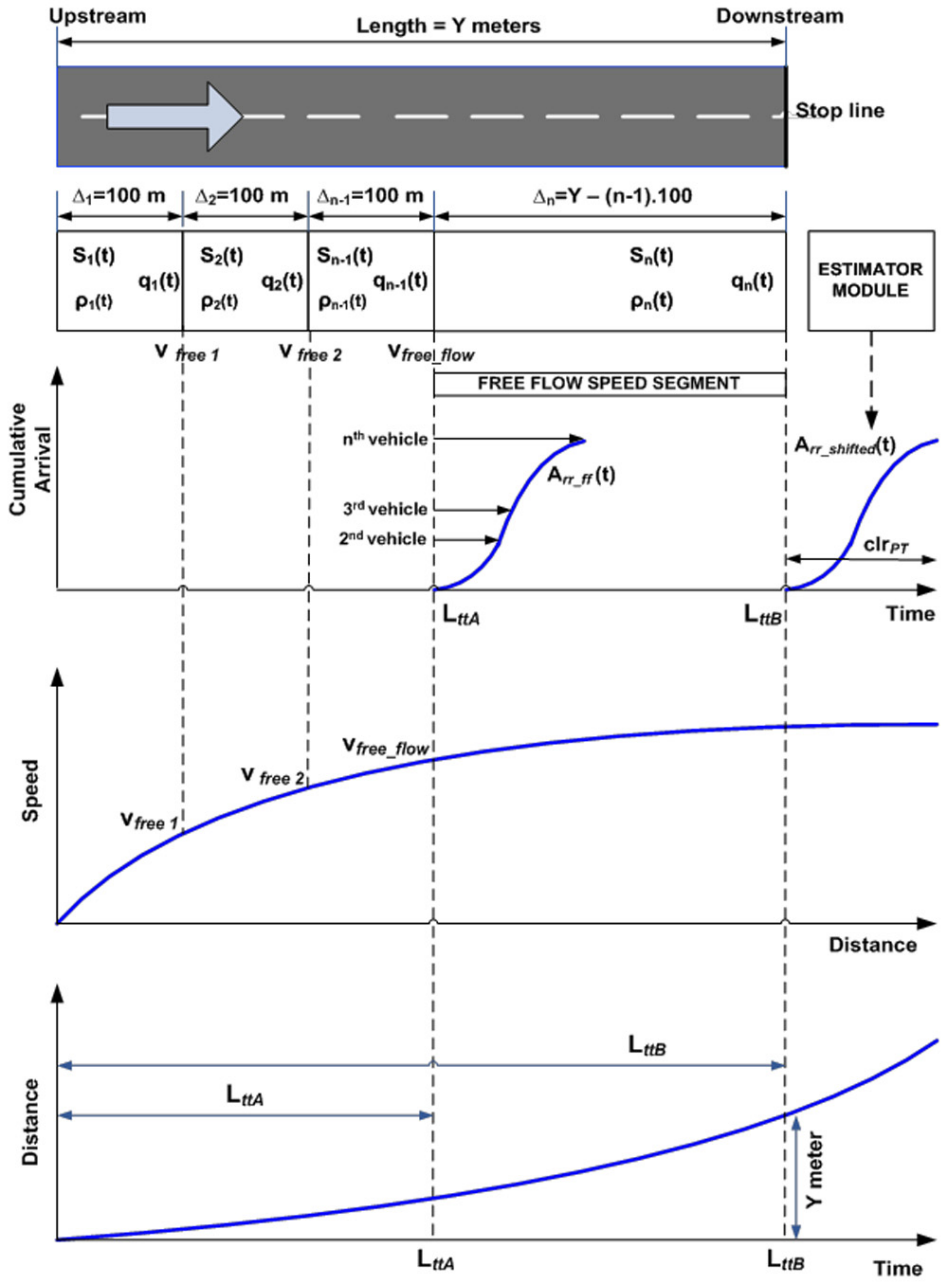
LWR-Integrated Model (LWR-IM). Fig 1 was previously published in [1] (corrected in [34]) and is subject to the license that applies to the original article. See the accompanying Expression of Concern for more information.

The proposed LWR-IM model (see Fig 1) is represented here to enable our readers to have an understanding of the underlying theoretical framework of the model previously published in [1]. The urban signalized arterial is described into segments of different lengths and traffic flow is modeled using the LWR and described by the continuous Petri nets with variable speeds (VCPN) model. This notion of describing the LWR using the VCPN is adapted from [23, 24]. As the LWR-based VPCN requires vehicle speeds information in each of the segments, the Rakha model [22] which could describe the dynamics of a single vehicle is integrated into the LWR-IM to feed speed information into the VCPN. These speeds are derived from its speed versus distance curve of the lead vehicle (see Fig 1). The Rakha model also provides travel time information (derived from its distance versus time curve) to the estimator for time-shifting the arrival curve obtained from the VCPN for input-output analysis at the targeted intersection downstream. The LWR-IM (i.e. the LWR based VCPN and Rakha models) are implemented using MATLAB coding. The functionalities of the LWR-IM can be explained as follows:

- Fig 1 shows an urban arterial that has been segmented. Each segment contains information of average speed *S*(t), density *ρ*(t) and flow rate *q*(t). The reader can refer to [1] on how the VCPN [23, 24] was used to describe the segmented arterial. Fig 1 also shows a typical speed versus time curve of the lead vehicle which is generated by the Rakha model [22]. It is used to define the maximum speed that a vehicle can attain in each segment of the motorway until it reaches free flow speed. Segments before a vehicle reaches free flow speed are 100 meters each whereas the length of the segment where most vehicles are traveling at free flow speed is the remaining distance until the downstream stop line. The ideal speed curve denotes the limited maximal speed *v*_*free i*_ in each arterial segment described by the VCPN that builds on continuum flow.
- The interaction and movement of subsequent vehicles in each segment are “constrained” by the conservation law and the fundamental relationship between flow, speed and density of the LWR based VCPN model.
- Rakha model also estimates the ideal travel time *L*_*tt*_ of the lead vehicle as it traverses down a motorway from an upstream stop line towards downstream stop line. This is illustrated in the distance versus time curve. *L*_*ttA*_ in the diagram denotes the travel time the lead vehicle takes to reach the free flow speed of the motorway whereas *L*_*ttB*_ is the travel time needed for the lead vehicle to reach downstream stop line.
- Arrival curve of vehicles can be obtained in the respective segments of the arterial. The first segment produces the input profile of the vehicles at the entrance. The arrival curve A_rr_ff_(t) obtained from the LWR-IM segment where all vehicles are assumed to have attained free flow speed describe behaviours of subsequent vehicles e.g. the time the second vehicle and the subsequent vehicles reaching this segment of the motorway relative to the *L*_*tt*_ of the lead vehicle.
- According to actuated performance measurement in [30–31], the arrival profile at free flow speed is time-shifted by a time constant, which is simply the distance of the advance detector divided by the free flow speed. Hence, it is justifiable to time-shift the arrival curve A_rr_ff_(t) obtained from the LWR-IM at free flow speed using the travel time of the lead vehicle *L*_*ttB*_.
- The estimator in Fig 1 is an algorithm developed in this work for time-shift the arrival profile produced by the LWR-IM according to the link travel time *L*_*ttB*_ of the lead vehicle. A typical cumulative platoon arrival profile is illustrated by A_rr_shifted_(t) in the diagram. The platoon width is denoted by clr_*PT*_ which defines the time needed for the platoon to clear the stop line.
- Traffic entering and leaving the motorway is determined by the signal timing plan e.g. phase change data for both upstream and downstream signals. The estimator utilizes the phase change data and downstream saturation flow rate to estimate a departure profile. Combining both arrived A_rr_shifted_(t) and departed vehicles, queues and delays can be estimated using the input-output analytical technique [1].

A few restrictions need to be mentioned about the scope of application of this model. The following are some of the restrictions:

- The model is applicable to uninterrupted coordinated control arterials with a range between 300 meters to 1 mile in length. Under uninterrupted conditions, platoons are assumed to progress downstream rather “smoothly” without splitting (due to a slow vehicle in the middle) compared to platoons moving in freeways.
- Vehicles overtaking each other and vehicles entering the platoon from the side streets are not considered and described by the model. This is due to the fact that vehicles overtaking are rather minimal in urban arterials as compared to freeways.

## Proposed Platoon Interaction Algorithms

Two platoons of vehicles may depart from the upstream intersection at different time instances because of different signal phasing. Both platoons progress downstream and may be served within the same green period. Within this time horizon, there is sufficient time and space for the first vehicle from the second platoon to close the gap with the last vehicle from the first platoon. Hence, both platoons seem to merge and can be considered as a single platoon.

**Fig 2:**
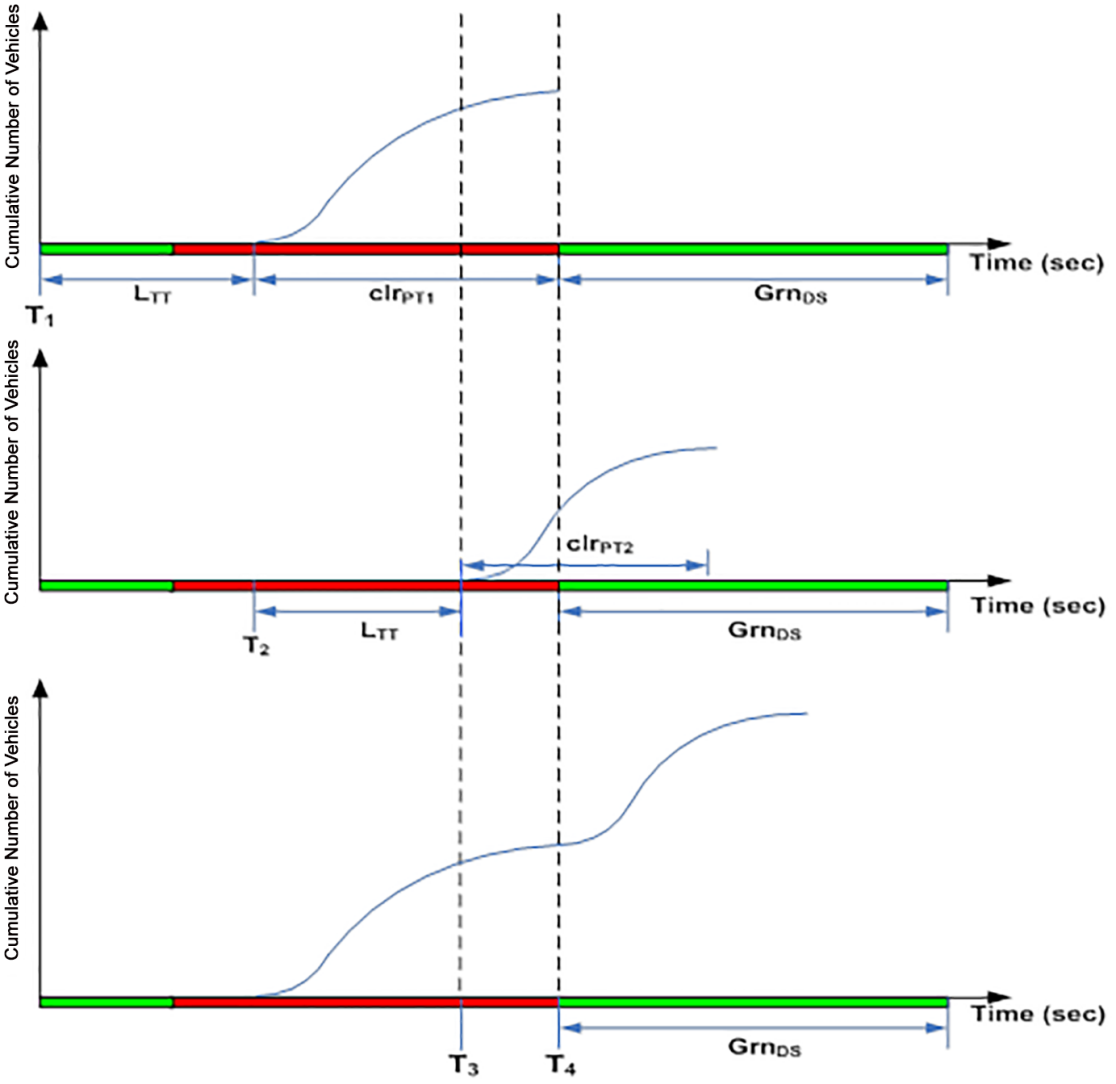
Scenario 1.

**Fig 3:**
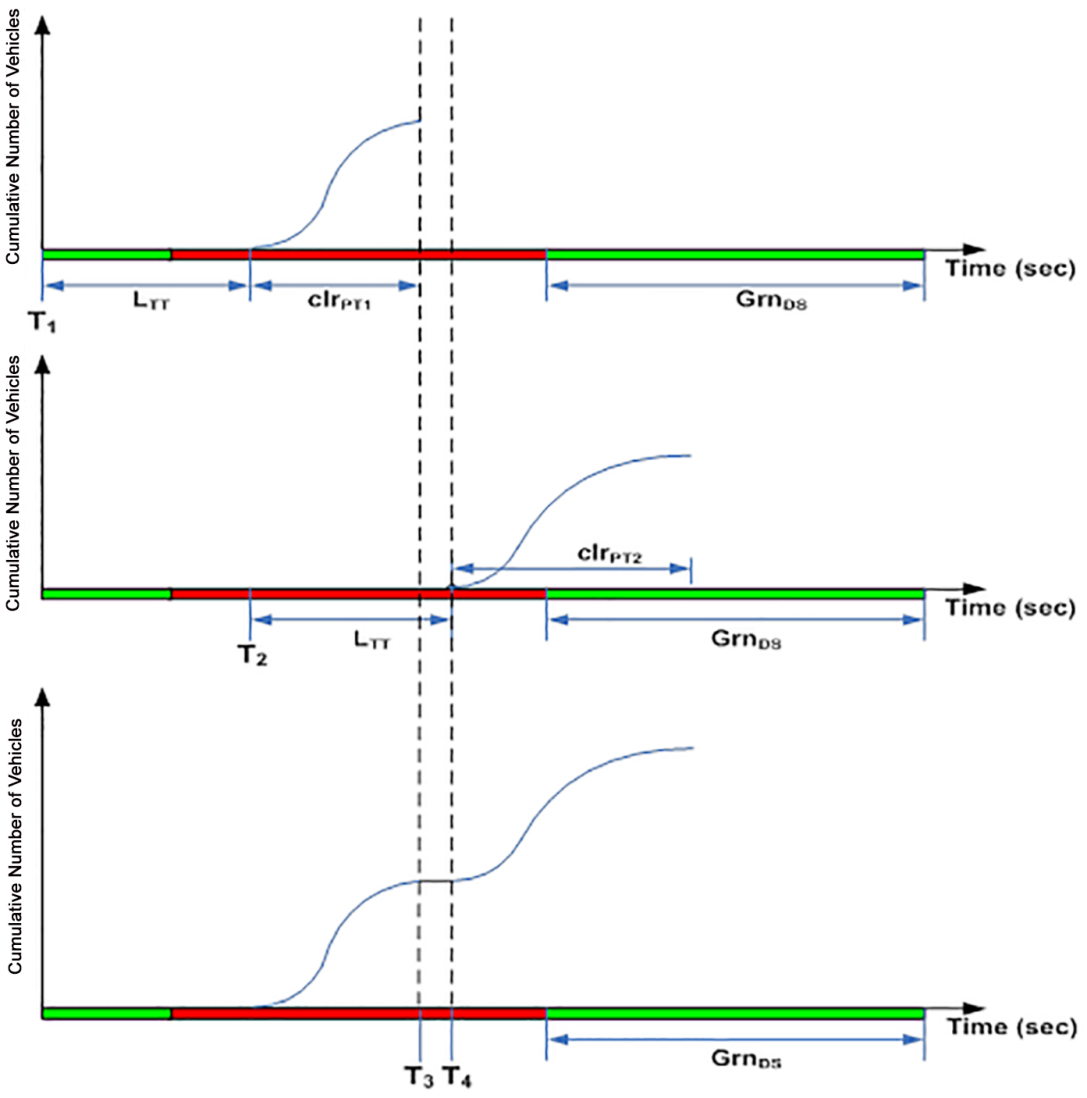
Scenario 2.

As an example, assuming a motorway receives two platoons of vehicles from the upstream signal as shown in Fig 2. The first platoon is released due to phase 1 of the upstream signal at time T_1_ where else the second platoon is released due to phase 2 at time T_2_. Both platoons move towards the downstream intersection with green period Grn_DS._ As both platoons move towards the downstream intersection and will be served within the same time horizon of the next green signal, both platoon profiles will be merged by the estimator and considered as a single platoon. Two scenarios of merging are evaluated by the estimator. The first scenario (Fig 2) happens when the second platoon is able to “catch up” with the first platoon. The second scenario (Fig 3) illustrates the second platoon “tailing” the first platoon within a finite temporal distance.

The *clr*_*PT1*_ in Fig 2 denotes the platoon clearance time for the first platoon of vehicles whereas the second platoon assumes a clearance time of *clr*_*PT2*_. The second platoon released at time T_2_ is able to close the gap with the first platoon where both platoons seem to overlap in the time window between T_3_ and T_4_. This shows that the first vehicle from the second platoon seems to be able to “catch up” with the last vehicle of the first platoon at close proximity. The second platoon is blocked and delayed by the first platoon by (T_4_-T_3_) seconds and join the rear of the first platoon at T_4_.

In contrast, scenario 2 in Fig 3 shows a “tailing” condition. Both platoons are released from the upstream intersection at time T_1_ and T_2_ respectively. The first platoon of vehicles has a clearance time *clr*_*PT1*_ whereas the second platoon assumes a clearance time of *clr*_*PT2*_. However, there is no overlap in the time window T_3_ to T_4_. This is considered a “tailing” condition where the first vehicle from the second platoon tails the last vehicle from the first platoon at a temporal distance (T_4_-T_3_). Consequently, the second platoon is merged with the first at time T_4_ where the number of vehicles in the time window is a constant (taking the value of the maximum number of vehicles from the first platoon).

For the purpose of emulating the effects of signal phases on platoon progressions at the real-world intersection, an algorithm is developed in the estimator module to emulate the platoon dynamics described in Figs 2 and 3 and performed merging of platoons according to the following algorithm:

**         If** T_2_ + L_tt_ *<* T_1_ + L_tt_ +*clr*_*PT1*_ **then**

**             ***Merge anterior of platoon 2 to the rear of platoon 1* at T_1_ + L_tt_ + *clr*_*PT1*_

**End If**

**         If** T_2_ + L_tt_ *>* T_1_ + L_tt_ +*clr*_*PT1*_ **then**

**             ***Extend the platoon 1 profile from* T_1_ + L_tt_ +*clr*_*PT1*_ *to* T_2_ + L_tt_

**             ***Next*, *merge anterior of platoon 2 to the rear of platoon 1* at T_2_ + L_tt_

**End If**

Even though platoons may be merged due to differing clearance and arrival times, the combined profiles can still be analyzed using the input-output analysis technique outlined in [1]. A detailed algorithm that describes merging behaviors of platoons is provided for in S1 Algorithm.

The S1 Algorithm (LinkModelMJ) describes the LWR-IM in terms of two moving platoons released from an upstream intersection at different time instance, i.e. US_*1*, *s*, *d*_ and US_*2*, *s*, *d*_ respectively. Line (4) of the algorithm obtains speed and travel time information from the Rakha model. Line (5) to (21) set each segment speed to the link free flow speed *v*_*free flow*_ if the segment speed exceeds the free flow speed in the link. The first platoon is released at time US_*1*, *s*, *d*_ with the number of vehicles denoted as *numOfVehicles1* whereas the second platoon is released at time US_*2*, *s*, *d*_ with *numOfVehicles2* number of vehicles. The arrival profiles (*arrProfile1* and *arrProfile2*) and clearing time (*clr*_*PT1*_ and *clr*_*PT2*_) of both platoons are obtained from the VCPN model (refer to line (25) to (39)). Line (41) to (43) implement platoon merging of *arrProfile1* and *arrProfile2* based on scenario 1 whereas line (45) to (48) implement platoon merging based on scenario 2 as prescribed in the previous section. The merged platoon clearing time is denoted as PCT in line (49). Subsequently, the merged platoon arrival profile is analyzed using the input-output method of the *IOAnalysis* module in line (50) to produce residue queue Q_r_, queue at begin green Q_s_ and total delay TD. Line (55) to (65) calculates TD, Q_r_, PCT, Q_s_ and *vehiclesToDownstream* when there are no vehicles entering the link. They are calculated based on the existing queue Iq_*s*, *d*_ in the link.

## Model Application and Validation Methods

### Model Application in Real-world Traffics

The proposed LWR-IM is applied to a class I arterial located in the city of Shah Alam, Malaysia and a class II arterial located in the city of Klang, Malaysia. Class I arterial can be defined as arterial having a length ranging from 800 m to 1609 m with a signal density of less than two signalized intersections per mile. On the other hand, class II arterial has length ranging from approximately 360 m to 800 m with a signal density of 2 to 4.5 signals per mile. For class I arterial, the free flow speed ranges from 45 to 55 mi/h (72.4 km/h to 88.5 km/h) whereas free flow speed in class II arterials ranges from 35 to 45 mi/h (56.32 km/h to 72.4 km/h). Hence, in this work simulated queues from the LWR-IM are validated with real traffic data collected from these locations.

#### Class I Arterial

**Fig 4:**
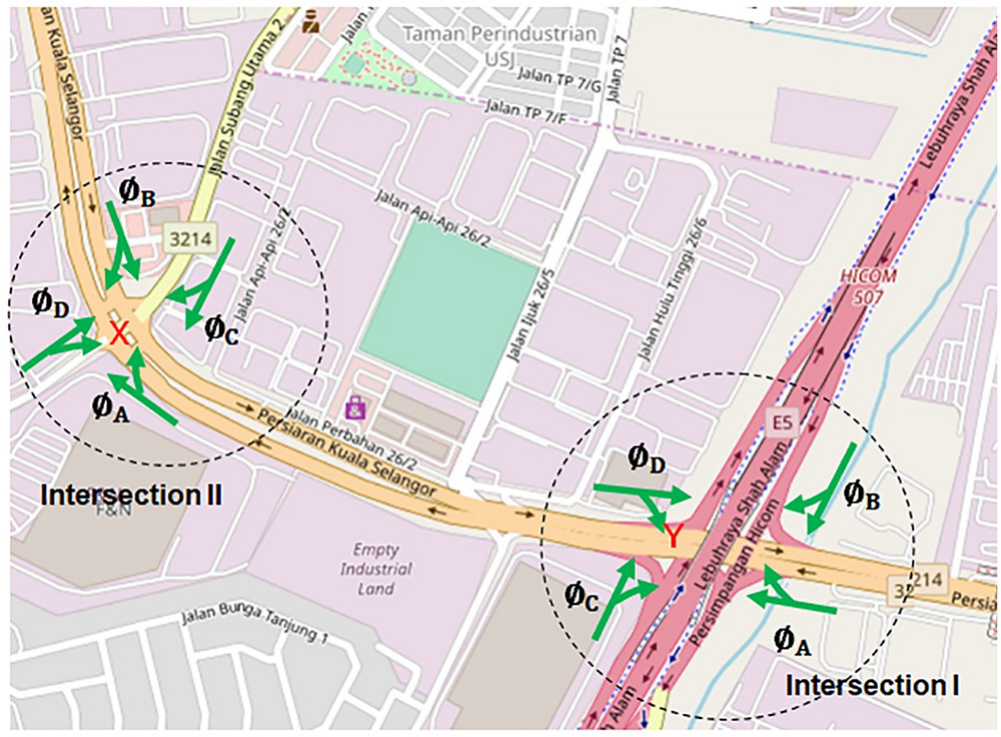
Real-world fixed-time traffic systems in Shah Alam, Malaysia, on Open Street Map [32]. This image was replaced when the accompanying Expression of Concern notice was published; see the notice for more information.

In this section, the LWR-IM is applied to a class I real-world fixed-time traffic system which is situated in the city of Shah Alam, Malaysia (Fig 4). The real traffic system consists of two intersections namely intersection I and II with their respective input and output links. Fig 4 also shows information about the phase transitions of the fixed-timed traffic lights at both intersections. Both traffic signal phase transitions start with phase A (Ø_A_), followed by phase B (Ø_B_), then phase C (Ø_C_) and finally, phase D (Ø_D_). Table 1 shows the green period of each phase with intersection I having a cycle time of 250 s, whereas intersection II operates at a cycle time of 220 s. Persiaran Kuala Selangor which has a length of 890 m is the class I arterial that connects both intersections. We are interested to simulate traffic dynamics that move along this arterial as it starts from the intersection I towards intersection II.

**Table 1:** Green time period for Intersection I and II.

Intersection	Ø_A_	Ø_B_	Ø_C_	Ø_D_
**I**	70 s	45 s	65 s	70 s
**II**	120 s	40 s	25 s	35 s

Performance criteria for the fixed-time intersection II in Fig 4 such as residue queues and queues at begin of green at location X which is the downstream stop line where vehicles stopped before turning right or going straight. The simulation is based on real traffic inputs collected from the intersections. Simulated queues are compared with real data collected from the intersections to validate the feasibility of the LWR-IM.

**Fig 5:**
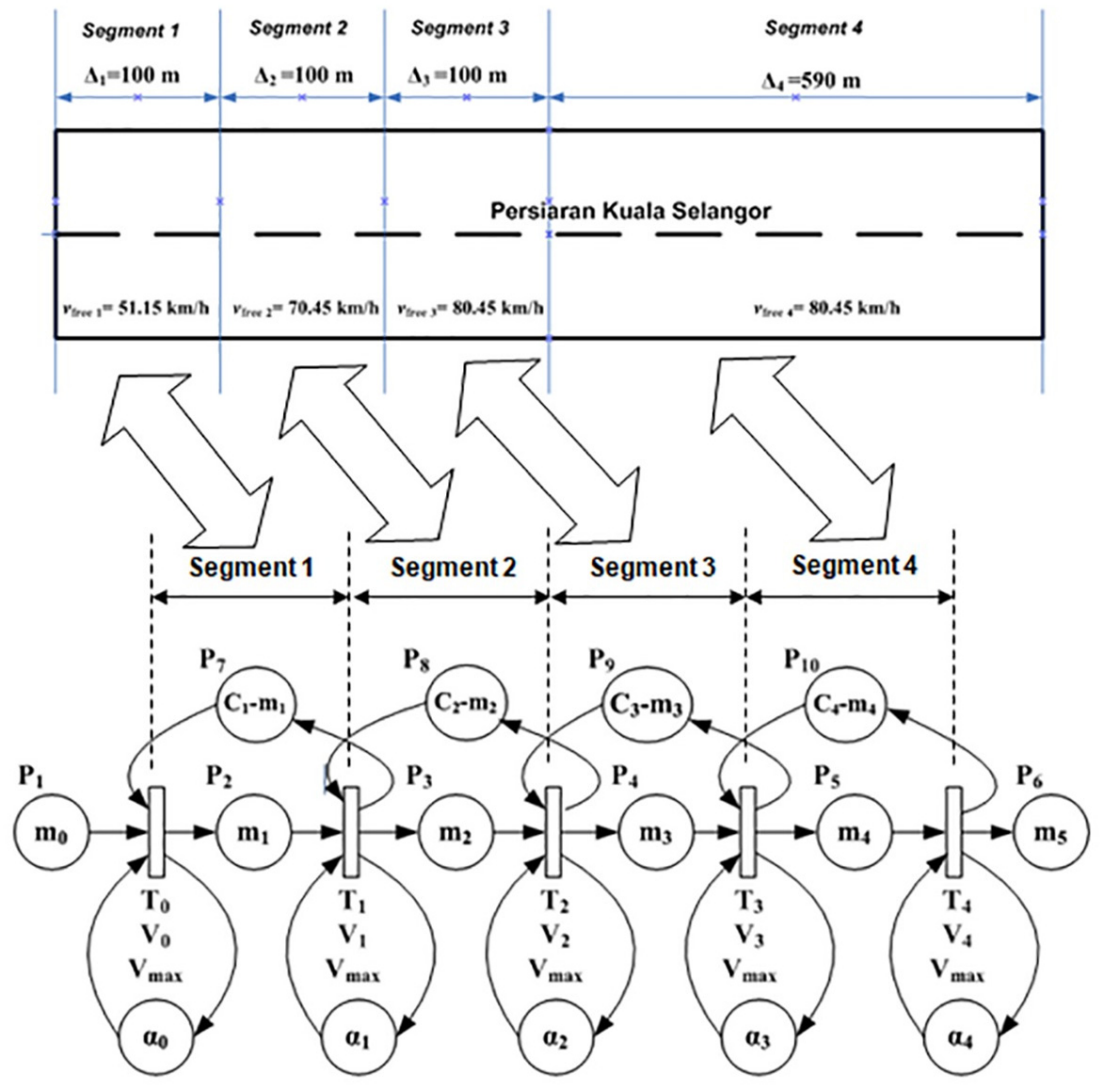
VCPN model of Persiaran Kuala Selangor. Adapted from models previously developed by Tolba et al. (see Figure 6 of [23] and Figure 9 of [24]).

**Fig 6:**
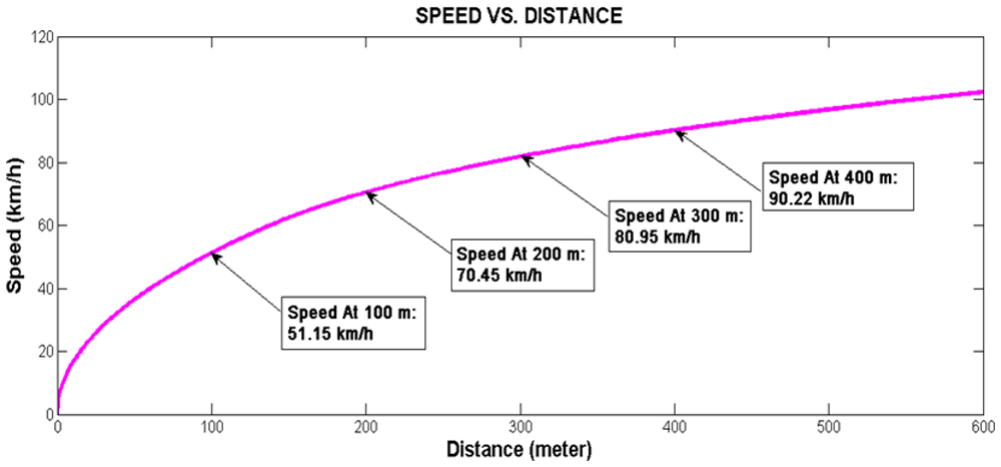
Speed versus distance plot.

Similar to our model application in [1]; Persiaran Kuala Selangor can be segmented into four segments as shown in Fig 5. The first three segments have similar length of 100m each, whereas the fourth segment assumes the remaining length of the arterial i.e. 590m. Fig 5 also shows the VCPN model developed in [23–24] that describes the traffic flow along Persiaran Kuala Selangor. The number of vehicles ready to depart from upstream intersection is indicated by marking m_0_. Markings in m_1_, m_2_, m_3_ and m_4_ represent the number of vehicles in segment 1, 2, 3 and 4 of Persiaran Kuala Selangor respectively. Markings in m_5_ represent the number of vehicles that reached the downstream stop line at location X. Markings C_1_-m_1_, C_2_-m_2_, C_3_-m_3_ and C_4_-m_4_ each represent the available capacity in each segment. A typical free flow speed of class I arterial is 80.45 km/h. According to the graph generated by the Rakha model in Fig 6, it is reasonable to assume that the lead vehicle achieves this speed at 300 m downstream and continue to move beyond 300 m with the same speed. Hence, the parameters of the VCPN can be calculated and summarized in Table 2. The maximum occupancy length of a vehicle is assumed to be 6.6 m.

**Table 2:** Traffic parameters of VCPN (Persiaran Kuala Selangor).

Segment	1	2	3	4
Δ_i_ (m)	100	100	100	590
*v*_*free i*_ (m/s)	14.21	19.57	22.34	22.34
V_max i_ (1/s)	0.1421	0.1957	0.2234	0.0378
C_i_*-*m_i_(veh)	15.15	15.15	15.15	89.39
*α*_*i*_(*t*) (veh)	3.78	3.78	3.78	22.35

Real traffic inputs into the arterial are collected via video recording for a period of one hour from 1245 to 1345. As the motorway of Persiaran Kuala Selangor consists of 3 lanes; the total volume of vehicles entering this arterial during each cycle is divided by 3. Thus, the number of vehicles entering each lane from upstream signal phases was observed for 16 consecutive signal timing cycles (approximately an hour). The number of vehicles in each lane denotes the initial marking m_0_ of the VCPN model.

Table 3 shows the tabulation of the observed traffic input volume per lane at Persiaran Kuala Selangor. The inputs originated from two incoming platoons, namely platoon A and platoon B which are released by upstream Ø_A_ and Ø_B_ respectively. The number of vehicles in platoon A and platoon B is considered as the initial marking m_0._ The total queues of Q_s_ and Q_r_ at downstream location X are also observed. Hence, queues per lane are obtained by dividing total queues by 3.

**Table 3:** Traffic volumes per lane for VCPN.

Cycle	Platoon A (vehs)	Platoon B (vehs)
**1**	33	10
**2**	0	0
**3**	34	11
**4**	31	10
**5**	30	10
**6**	29	6
**7**	33	14
**8**	39	11
**9**	36	13
**10**	31	12
**11**	0	0
**12**	36	11
**13**	35	13
**14**	31	10
**15**	38	19
**16**	35	12

These traffic inputs are regulated by the signal timing plan which is coded in MATLAB for 16 consecutive cycles. The cumulative vehicle arrival profile is predicted by the LWR-IM model from a cycle to cycle basis. Next, the estimating module (mentioned in Fig 1) performs the following:

- Time-shift the arrival profiles according to the *L*_*tt*_ obtained from the Rakha model.
- Merging of arriving profiles according to platoon merging methods in section 3.
- Calculate Q_s_ and Q_r_ at the downstream stop line (location X) using the input-output analytical method. Finally, simulated Q_s_ and Q_r_ are compared with real Q_s_ and Q_r_ collected from the intersection.

#### Class II Arterial

**Fig 7:**
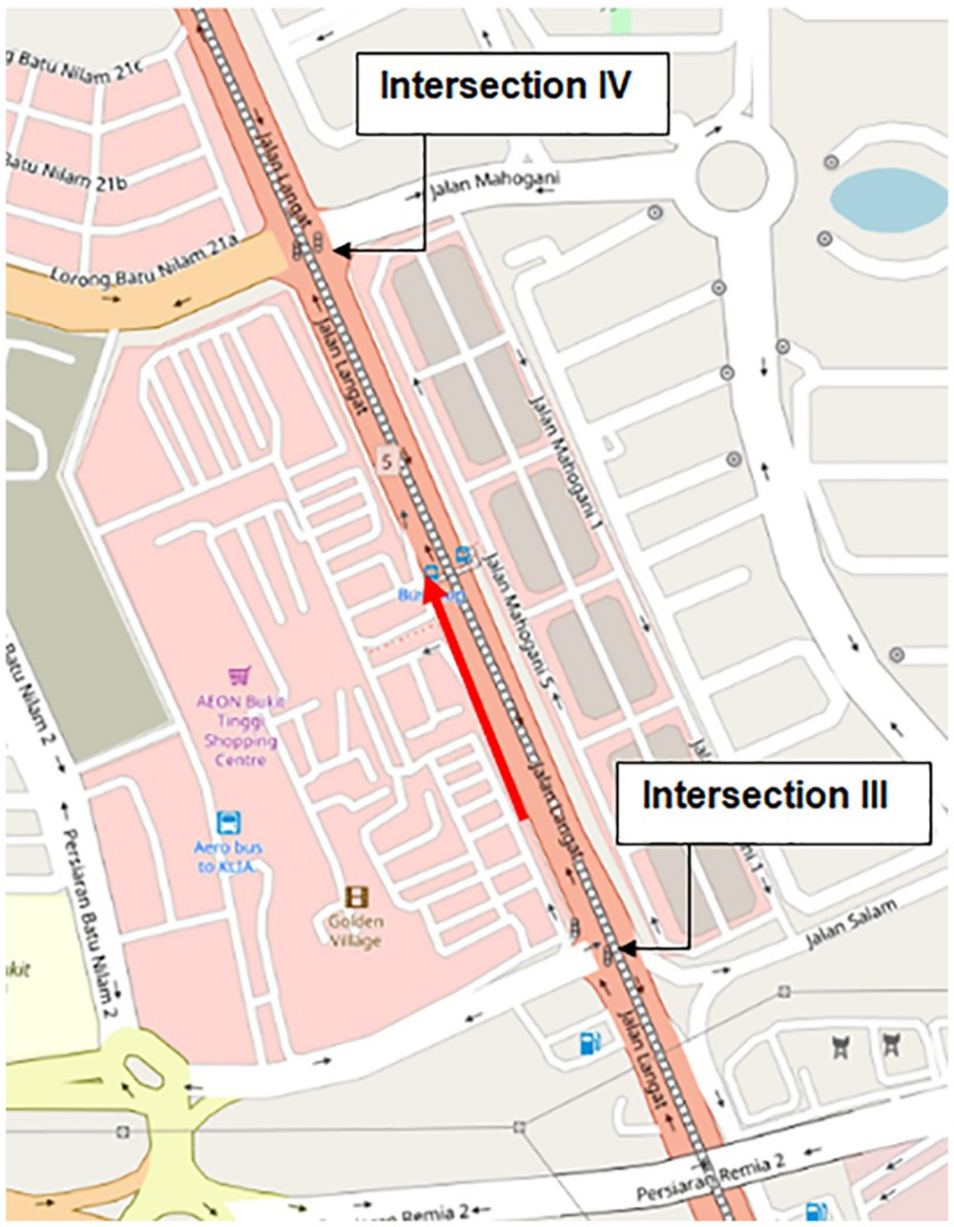
Fixed-time traffic systems at Jalan Langat on Open Street Map [33]. This image was replaced when the accompanying Expression of Concern notice was published; see the notice for more information.

Similar validation conducted previously for class I arterial is also conducted with another class II arterial, namely Jalan Langat in the city of Klang. Fig 7 shows the layout of Jalan Langat (3 lane arterial) which is 550 meters in length. The direction of traffic is indicated by the red arrow. The LWR-IM is applied to model traffic dynamics along Jalan Langat starting from intersection III towards intersection IV. The characteristics of each intersection such as the green period that give the right of way for the incoming platoons (at intersection III) and the outgoing platoons (at intersection IV), the cycle time and number of signal phases of both intersections are listed in Table 4.

**Table 4:** Characteristics of Intersections.

Intersection	Cycle Time	Green Period	Number of signal phases
**III**	115	53	3
**IV**	215	85	4

The VCPN that describes the traffic flow along Jalan Langat for this location takes the exact form of the model for Persiaran Kuala Selangor. However, free flow speed is set at 60 km/h. This is in the range of the free flow speed outlined in HCM for class II arterials. The VCPN parameters are listed in Table 5. However, the lead vehicle reaches free flow speed at 200 meters and continues to move at the same speed until it reaches the downstream stop line.

**Table 5:** Traffic parameters of VCPN (Jalan Langat).

Segment	1	2	3	4
Δ_i_ (m)	100	100	100	250
*v*_*free i*_ (m/s)	14.21	16.67	16.67	16.67
V_max i_ (1/s)	0.1421	0.1667	0.1667	0.067
C_i_*-*m_i_(veh)	15.15	15.15	15.15	37.88
*α*_*i*_(*t*) (veh)	3.78	3.78	3.78	9.47

Traffic volume entering from intersection III is observed and tabulated manually. Table 6 shows observed traffic input volume per lane (released from intersection III) for 16 cycles of intersection IV. Intersection III only releases a single platoon during each cycle. These entry volumes are considered as initial marking m_0_. The LWR-IM is implemented for 16 signal cycles (approximately an hour) with an initial queue of 21 vehicles at intersection IV. Finally, simulated Q_s_ and Q_r_ from the LWR-IM are compared with real Q_s_ and Q_r_ observed at intersection IV.

**Table 6:** Traffic input volume per lane for Jalan Langat.

Cycle	Observed traffic volume (per lane)	Cycle	Observed traffic volume (per lane)
**1**	3	**9**	25
**2**	27	**10**	33
**3**	29	**11**	30
**4**	23	**12**	7
**5**	25	**13**	18
**6**	28	**14**	11
**7**	37	**15**	34
**8**	28	**16**	25

### Quantitative Model Validation Approach

The validation involves comparison of simulated queues and real queues observed at both class I and class II arterials mentioned in section 4.1. The authors employ the t-test statistical analysis approach for this comparison to evaluate any significant difference between simulated and real queues. The procedure is outlined as follows:

Step 1: The level of significance (LOS), LOS = 0.1 is chosen for this test.
Step 2: Simulation runs are conducted and the queue produced in each cycle of the simulation is tabulated. Observed queues are also tabulated and the mean absolute errors are calculated.
Step 3: A two-sample t-test is conducted using statistical facilities in the Microsoft Excel. The p-value is computed in this test to ascertain the type of hypothesis achieved by the model. The null hypothesis depicts that the simulated and real queues are the same whereas the alternative hypothesis portrays otherwise.
Step 4: If the p-value is less than the pre-defined LOS, then the null hypothesis is rejected and the alternative hypothesis is accepted, indicating that the model is invalid. On the other hand, if the p-value is more than the pre-defined LOS, the null hypothesis is accepted, indicating that the model is valid for the chosen performance indices (i.e. queues).
Step 5: If the null hypothesis is accepted, the model can be concluded as valid for its intended purpose. Otherwise, the model is invalid for that purpose.

## Simulation and Model Validation Results

**Fig 8:**
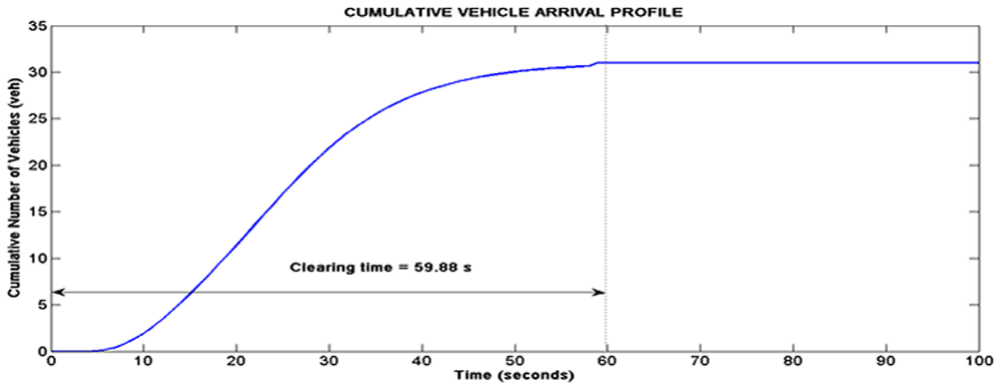
Arrival profile of platoon A.

**Fig 9:**
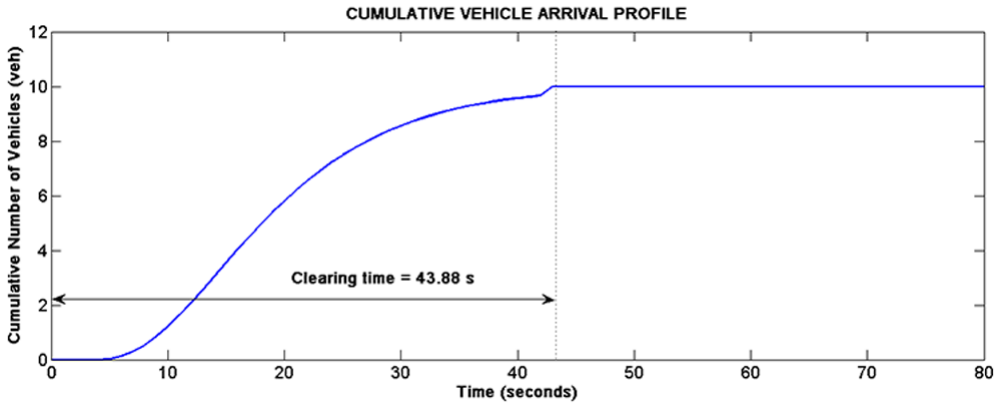
Arrival profile of platoon B.

This section presents the validation of simulation results with data from real world traffic in both of the class I and class II arterials. Simulation for cycle 4 of intersection II of Persiaran Kuala Selangor will be used to illustrate the functions of the platoon interaction algorithm. According to Table 3, the total volume of vehicles per lane entering Persiaran Kuala Selangor is 31 vehicles and 10 vehicles, which are released from phase A and B respectively. Based on the input of 31 vehicles from platoon A, the LWR-IM produced the cumulative arrival profile of platoon A as shown in Fig 8. On the other hand, Fig 9 shows the arrival profile simulated based on 10 vehicles from platoon B. The graphs in Figs 8 and 9 show, respectively the clearance time for both platoons of vehicles where *clr*_*PTA*_ = 59.88 s and *clr*_*PTB*_ = 43.88 s.

**Fig 10:**
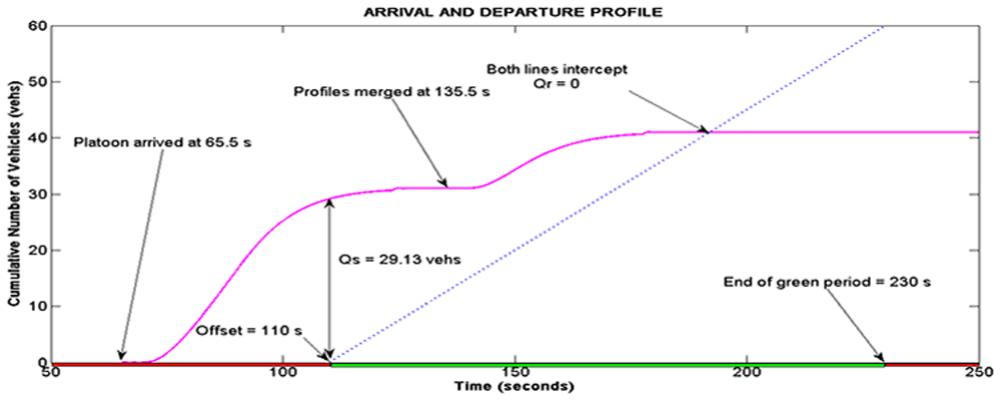
Arrival and departure profile at Intersection II.

The implementation is conducted using a time clock that starts at *t* = 0 s with a time step of 1 s. At cycle 4, the platoon A is released at *t* = 550 s, whereas platoon B is released at *t* = 620 s respectively from intersection I. The time instance the downstream intersection turns green is at *t* = 660 s. These time parameters are normalized with respect to *t* = 0 in Fig 10 to enable easier analysis. Hence, upon normalization, platoon A is released at *t* = 0 s, whereas platoon B is released at *t* = 70 s. The *L*_*tt*_ of Persiaran Kuala Selangor simulated by the Rakha model is approximately 65.48 s. Hence, the arrival time of platoon A at the downstream intersection is approximately 65.48 s, whereas platoon B will reach downstream at 135.48 s.

The time parameters of both platoons depict a scenario 2 platoon interaction. Thus, the anterior part of the arrival profile in Fig 8 is merged to the rear of the profile in Fig 9 as shown in Fig 10. The merging occurs at 135.48 s. Fig 10 also illustrates calculation of Q_s_ and Q_r_ at location X (intersection II) based on this merged profile. The estimator estimates a departure profile at the downstream intersection based on the phase offset of 110 s and the downstream green period and cycle time which is set at 120 s and 220 s respectively.

The simulated result in Fig 10 shows that the arrival profile reaches the downstream stop line at 65.48 s. The graph also shows 29.13 vehicles formed the queue at the beginning of the green period. However, all the vehicles cleared the intersection stop line at the end of the green period. Therefore, there is no residue queue. Similar simulation is repeated for the other cycles.

Table 7 shows the tabulation of real and simulated queues at begin of green Q_s_ and residue queues Q_r_ over a span of 16 cycles of the timing plan of intersection II of Persiaran Kuala Selangor. Simulated Q_s_ closely agree for cycle 1, 4, 5, 12, 13 and 15. Simulated Q_s_ are higher than real Q_s_ at cycle 2 and 3. On the other hand, simulated Q_s_ are generally lower than real data from cycle 6 to 11. The highest discrepancy is observed at cycle 9 with a difference of 13.9 vehicles whereas the lowest discrepancy is observed at cycle 1. Table 7 also displays the tabulated results of Q_r_ for Persiaran Kuala Selangor. Simulated residue queues are in closer agreement for cycle 3, 5, 10, 11 and 12. The highest discrepancy is observed at cycle 15 with a difference of 3.57 vehicles whereas the lowest discrepancy is observed at cycle 3, 5, 10, 11 and 12 with zero differences.

**Table 7:** Simulated and real queues of Persiaran Kuala Selangor.

Cycle	Queue at begin of green Q_s_			Residue queue Q_r_		
	Real	Simulated	Absolute Difference	Real	Simulated	Absolute Difference
**1**	1.00	1.00	0.00	35.33	31.58	3.75
**2**	35.33	42.64	7.31	1.00	0.00	1.00
**3**	28.33	34.21	5.88	0.00	0.00	0.00
**4**	29.66	29.13	0.53	1.00	0.00	1.00
**5**	6.66	5.69	0.97	0.00	0.00	0.00
**6**	4.00	0.00	4.00	4.00	0.55	3.45
**7**	4.00	0.55	3.45	16.00	13.53	2.47
**8**	16.00	14.24	1.76	6.66	3.36	3.30
**9**	28.66	14.76	13.9	30.66	31.89	1.23
**10**	47.00	44.86	2.14	0.00	0.00	0.00
**11**	38.66	36.59	2.07	0.00	0.00	0.00
**12**	35.33	35.16	0.17	0.00	0.00	0.00
**13**	16.00	16.43	0.43	1.00	0.00	1.00
**14**	4.00	0.00	4.00	1.50	0.00	1.50
**15**	0.00	0.00	0.00	10.00	13.57	3.57
**16**	10.00	14.26	4.26	3.33	1.15	2.18

Tabulated real and simulated Q_s_ and Q_r_ over a span of 16 cycles of the timing plan of intersection IV of Jalan Langat are shown in Table 8. The tabulated results reveal that simulated Q_s_ are closely in agreement with real Q_s_ for cycle 1, 3, 4, 5, 6, 7 and 15. However, simulated Q_s_ are generally higher than real Q_s_ for cycle 2, 8, 9 and 12. The highest discrepancy is observed at cycle 9 with a difference of 10.14 vehicles where else the lowest discrepancy is observed at cycle 7 with zero difference. On the other hand, simulated Q_r_ coincides with real Q_r_ for cycle 1, 8, 14, 15 and 16. However, simulated Q_r_ are found to be higher than real Q_r_ for cycle 2, 3, 4, 5, 6, 7, 9, 10 and 13. The highest discrepancy is observed at cycle 3 with a difference of 5.10 vehicles whereas the lowest discrepancy is observed at cycle 1 with a discrepancy of 0.05 vehicles.

**Table 8:** Simulated and real queues of Jalan Langat.

Cycle	Queue at begin of green Q_s_			Residue queue Q_r_		
	Real	Simulated	Absolute Difference	Real	Simulated	Absolute Difference
**1**	21	21.08	0.08	15	14.95	0.05
**2**	15	20.84	5.84	0	2.10	2.10
**3**	10	10.27	0.27	0	5.10	5.10
**4**	5	5.10	0.10	0	4.05	4.05
**5**	10	10.20	0.20	0	4.50	4.50
**6**	21	21.25	0.25	0	4.95	4.95
**7**	17	17.00	0.00	0	3.78	3.78
**8**	8	11.43	3.43	14	13.90	0.10
**9**	14	24.14	10.14	8	11.90	3.90
**10**	25	16.74	8.26	0	3.98	3.98
**11**	15	10.62	4.38	5	4.58	0.42
**12**	12	15.30	3.30	5	3.90	1.10
**13**	5	0.00	5.00	2	3.15	1.15
**14**	2	0.00	2.00	3	2.50	0.50
**15**	9	9.89	0.89	7	7.47	0.47
**16**	21	15.04	5.96	16	16.12	0.12

The two-sampled t-test performed on both real and simulated Q_s_ and Q_r_ of Persiaran Kuala Selangor produces p-values of 0.87 and 0.81 respectively (refer to Table 9). Thus, the null hypothesis can be accepted as these p-values are more than LOS = 0.1. This indicates that the simulation and the real-world data of Persiaran Kuala Selangor are not significantly different at LOS = 0.1 with respect to Q_s_ and Q_r_. The same conclusion also can be reached for Jalan Langat (refer to Table 9). The p-value for simulated and real Q_s_ of Jalan Langat is 0.98 whereas the p-value for simulated and real Q_r_ stands at 0.29. Since the null hypothesis cannot be rejected for both Persiaran Kuala Selangor and Jalan Langat at LOS = 0.1 for both Q_s_ and Q_r_, we believe that the LWR-IM is valid for its intended purpose.

**Table 9:** p-value and mean absolute error (MAE).

Location	p-value		MAE	
	Queue at begin green Q_s_	Residue queue Q_r_	Queue at begin green Q_s_	Residue queue Q_r_
**Persiaran Kuala Selangor**	0.87	0.81	3.18	1.53
**Jalan Langat**	0.98	0.29	3.13	2.27

The insignificant differences between simulated and real data concluded by the two-sampled t-test reveal the ability of the LWR-IM to simulate results that closely resemble real situation. This is also supported by the seemingly low mean absolute error (MAE) calculated using data in Tables 7 and 8 respectively. Table 9 shows that MAEs for real and simulated Q_s_ and Q_r_ of Persiaran Kuala Selangor are 3.18 and 1.53 vehicles respectively. Table 9 recorded MAEs of 3.13 and 2.27 for simulated and real Q_s_ and Q_r_ respectively in Jalan Langat. These low MAEs depict that at every signal cycle, the comparison of simulated and real data reported an average absolute error of between 1.53 and 3.16 vehicles.

## Conclusion

In this work, the authors proposed platoon interaction algorithms to the LWR-IM model to describe platoon interactions in urban arterials. Consequently, the work also aims to quantitatively validate the LWR-IM and its underlying algorithm with real-world data. The LWR-IM and the proposed algorithm has been applied and implemented in a real-world class I and class II arterials. Validation of the model is conducted with real queue data to ascertain: (i) its ability to simulate queues that closely resemble actual queues and (ii) the viability of the platoon interaction algorithms of producing accurate and utilizable platoon arrival profile. The results from the t-test with p-values range from 0.29 and 0.98 for four sets of comparisons between real and simulated queues at both class I and class II arterials evidently prove that both criteria have been appropriately validated as real and simulated queues did not differ significantly from each other. In addition, the statistical evaluations via the mean absolute error reveal low absolute errors of vehicle queues. The analyses have proven that the LWR-IM and its underlying algorithms are valid and feasible for its intended purpose.

## Supporting Information

S1 Algorithm (DOCX)

S1 File MATLAB Plot for Queue at Begin Green at Persiaran Kuala Selangor. (FIG)

S2 File MATLAB Plot for Residue Queue at Persiaran Kuala Selangor. (FIG)

S3 File MATLAB Plot for Queue at Begin Green at Jalan Langat. (FIG)

S4 File MATLAB Plot for Residue Queue at Jalan Langat. (FIG)

S5 File JPEG Image for Queue at Begin Green at Persiaran Kuala Selangor. (JPG)

S6 File JPEG Image for Residue Queue at Persiaran Kuala Selangor. (JPG)

S7 File JPEG Image for Queue at Begin Green at Jalan Langat. (JPG)

S8 File JPEG Image for Residue Queue at Jalan Langat. (JPG)

S9 File Statistical Analysis. TTest and Mean Absolute Error calculations.
(XLSX)
