# Expression of Concern: Platoon Interactions and Real-World Traffic Simulation and Validation Based on the LWR-IM

**DOI:** 10.1371/journal.pone.0306213

**Published:** 2024-07-09

**Authors:** The PLOS ONE Editors

## Notice of republication

After publication of this article [1], concerns were raised about similarities of this work to previously published articles [2–6], reuse of equations and figures from those articles, and whether these related works were sufficiently discussed in the article so as to place the new work in the *PLOS ONE* article [1] into context.

To address these issues, the article [1] was republished at the time of this notice’s publication to incorporate the following changes:

- The Introduction has been updated to explain how this article builds upon previous work [2–6] and how it is distinct from the work reported in [2] (subsequently corrected in [3]).
- The LWR-IM Model section in [1] serves as a recap of previous work [2, 3], which cited work by Tolba et al. [4, 5] (as references 17 and 18 of [2]). While this section of [1] cited the authors’ previous article [2, 3], it did not directly attribute the VCPN model to the original publications [4, 5]. Furthermore, the original publication for the Rakha model [6] was cited in the Introduction but was not cited later in the article so as to provide clear attribution where the published model was used. The authors apologize for these omissions. In the revised version [1], text has been added to the LWR-IM Model section to provide clear attribution to [4–6] (references 22–24 in [1]).
- Equations 1 and 2 in the original published version of [1] were previously published in [5] as equations 26 and 25, respectively. These equations and the two paragraphs preceding them were removed in the updated version of [1].
- Fig 1 was published previously in [2, 3] (reference 1 in [1]). A citation to the original open access article has been added to the figure legend.
- Figs 2 and 3 were similar to content published previously in [2–5]. Figs 2 and 3 are replaced with a new diagram in Fig 5, the legend of which specifies the model was adapted from those previously published in [4, 5].
- Figs 6 and 8 in the original version of [1] were adapted from map images subject to license restrictions. There was an error in Fig 8, which showed the incorrect intersection along Jalan Langat. In the republished version, these figures have been replaced with images of the correct locations prepared using Open Street Map, and have been renumbered as Figs 4 and 7, respectively.
- The figures have been renumbered in the republished article to account for the above changes.

The *PLOS ONE* Editors consider that the above amendments resolve the concerns regarding attribution. Please download this article again to view the correct version.

The primary data for this study were not included with the article, contrary to the Data Availability statement. The raw video recordings of the traffic at both locations are no longer available. The real traffic observations obtained from these video recordings were tabulated in Tables 3, 6, 7 and 8 [1]. At the time of republication the original article was updated to include the other available data and analysis files in S1-S9 Files. As the raw video recordings are now unavailable, this article [1] does not comply with the PLOS Data Availability Policy. The *PLOS ONE* Editors, therefore, issue this Expression of Concern to inform readers of this data availability issue.

## References

[1] Ng KM, Reaz MBI (2016) Platoon Interactions and Real-World Traffic Simulation and Validation Based on the LWR-IM. PLoS ONE 11(1): e0144798. 10.1371/journal.pone.0144798 26731745 PMC4701377

[2] Ng KM, Reaz MBI (2015) An Integrated Approach for Platoon-based Simulation and Its Feasibility Assessment. PLoS ONE 10(3): e0114406. 10.1371/journal.pone.0114406 25785693 PMC4364894

[3] Ng KM, Reaz MBI (2024) Correction: An Integrated Approach for Platoon-based Simulation and Its Feasibility Assessment. PLoS ONE 19(VOL): e0306215. 10.1371/journal.pone.0306215 PMC4364894 25785693

[4] Tolba C, Lefebvre D, Thomas P, Moudni AE (2005) Continuous and timed Petri nets for the macroscopic and microscopic traffic flow modeling. Simulation, Modeling Practice and Theory13: 407–436. 10.1016/j.simpat.2005.01.001

[5] Tolba C, Lefebvre D, Thomas P, Moudni AE (2001) Continuous Petri nets models for the analysis of urban traffic networks. Proceedings of 2001 IEEE International Conference on Systems, Man and Cybernetics. pp. 1323–1328. doi: 10.1109/ICSMC.2001.973104

[6] Rakha H, Lucic I, Demarchi S, Setti J, Aerde VM (2001) Vehicle dynamics model for predicting maximum truck acceleration levels. Journal of Transportation Engineering 127(5): 418–425.

