# Supplementary material for: Platoon Interactions and Real-World Traffic Simulation and Validation Based on the LWR-IM
Source: PLoS One. 2016 Jan 5;11(1):e0144798. doi: 10.1371/journal.pone.0144798 (PMC4701377; doi:10.1371/journal.pone.0144798)
Supplement: S1 Algorithm — (DOCX) [file pone.0144798.s001.docx]

| **S1 Algorithm (LinkModelMJ)** | |
| --- | --- |
| 1:  2:  3:  4:  5:  6:  7:  8:  9:  10:  11:  12:  13:  14:  15:  16:  17:  18:  19:  20:  21:  22:  23:  24:  25:  26:  27:  28:  29:  30:  31:  32:  33:  34:  35:  36:  37:  38:  39:  40:  41:  42:  43:  44:  45:  46:  47:  48:  49:  50:  51:  52:  53:  54:  55:  56:  57:  58:  59:  60:  61:  62:  63:  64:  65:  66: | Obtain *numOfVehicles1*, *numOfVehicles2*, US*_1,s,d_*, US*_2,s,d_*  *capAbove300* = (*LinkLength – 300*)/6.6  *dischargeRate* = *saturationFlow/3600*  Obtain $v_{free 1}$, $v_{free 2}$, $v_{free 3}$, $v_{free 4}$and *L_TT_* from *Rakha model*  **If** $v_{free 1}$ >= $v_{free flow}$**then**  $v_{free 1}$= $v_{free flow}$  **End If**  **If** $v_{free 2}$ >= $v_{free flow}$**then**  $v_{free 2}$= $v_{free flow}$  **End If**  **If** $v_{free 3}$ >= $v_{free flow}$**then**  $v_{free 3}$= $v_{free flow}$  **End If**  **If** $v_{free 4}$ >= $v_{free flow}$**then**  $v_{free 4}$= $v_{free flow}$  **End If**  **If** *NumOfVehicles1* > 0 and *NumOfVehicles2* > 0 **then**  **If** *NumOfVehicles1* > 0 **then**  Call VCPN model  Obtain *arrProfile1* and *clr_PT1_*  **Else**  *arrProfile1* = null  *clr_PT1_* = 0  **End If**  **If** *NumOfVehicles2* > 0 **then**  Call VCPN model  Obtain *arrProfile2* and *clr_PT2_*  **Else**  *arrProfile2* = null  *clr_PT2_* = 0  **End If**  **If** US*_2,s,d_* + *L_tt_ <* US*_1,s,d_* + *L_tt_* +*clr_PT1_* **then**  Merge anterior of *arrProfile2* to the rear of *arrProfile1* at US*_1,s,d_* + *L_tt_* + *clr_PT1_*  **End If**    **If** US*_2,s,d_* + *L_tt_ >* US*_1,s,d_* + *L_tt_* +*clr_PT1_* **then**  Extend the *arrProfile1* from US*_1,s,d_* + *L_tt_* +*clr_PT1_* *to* US*_2,s,d_* + *L_tt_*  Merge anterior of *arrProfile2* to the rear of *arrProfile1*at US*_2,s,d_* + L_tt_  **End If**  PCT = length of merged profiles  Call *IOAnalysis*  Obtain Q_r_, Q_s_ and TD from *IOAnalysis*  *vehiclesToDownstream* = *NumOfVehicles1* + *NumOfVehicles1* + Iq*_s,d_* – Q_r_  **End If**  **If** *NumOfVehicles1* == 0 **and** *NumOfVehicles2* == 0 **then**  Q_s_ = Iq*_s,d_*  Calculate TD using Iq*_s,d_*  Q_r_ = Iq*_s,d_* – (*GreenDuration * dischargeRate*)  **If** Q_r_ <= 0 **then**  Q_r_ = 0  **End If**  PCT = 0  *vehiclesToDownstream* = Iq*_s,d_* - Q_r_  **End If**  Return Q_r_, TD, PCT, *vehiclesToDownstream* and Q_s_ |
