# Supplementary figures and images for: Platoon Interactions and Real-World Traffic Simulation and Validation Based on the LWR-IM
Source: PLoS One. 2016 Jan 5;11(1):e0144798. doi: 10.1371/journal.pone.0144798 (PMC4701377; doi:10.1371/journal.pone.0144798)

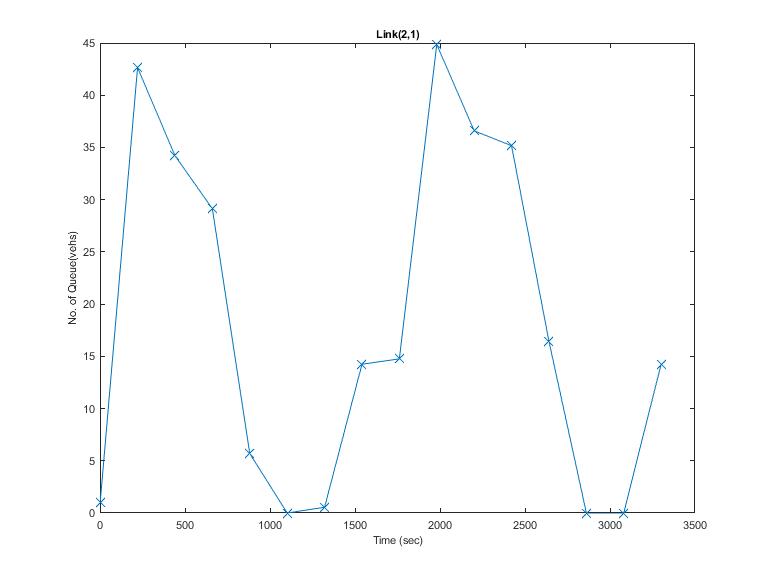

Supplement: S5 File — (JPG) [file pone.0144798.s006.jpg]

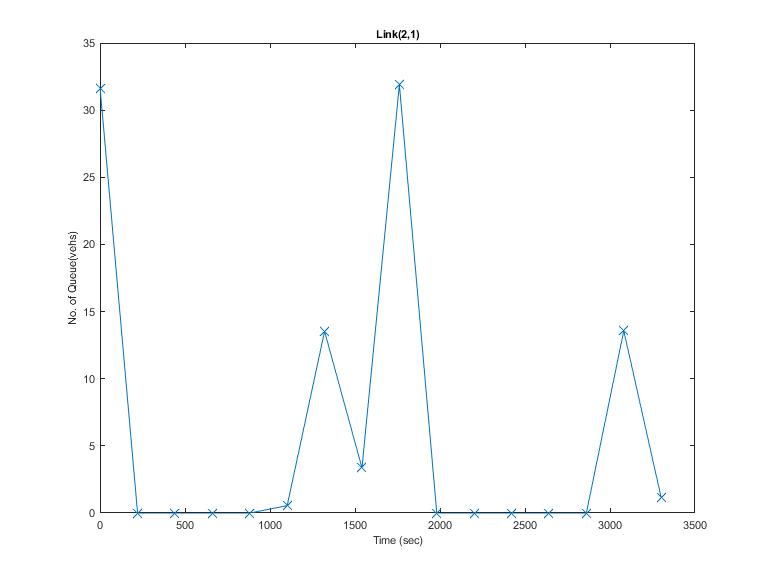

Supplement: S6 File — (JPG) [file pone.0144798.s007.jpg]

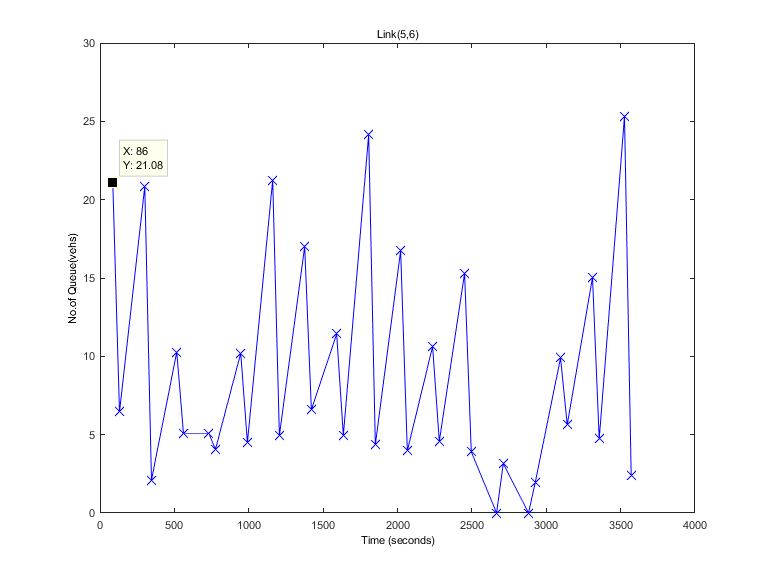

Supplement: S7 File — (JPG) [file pone.0144798.s008.jpg]

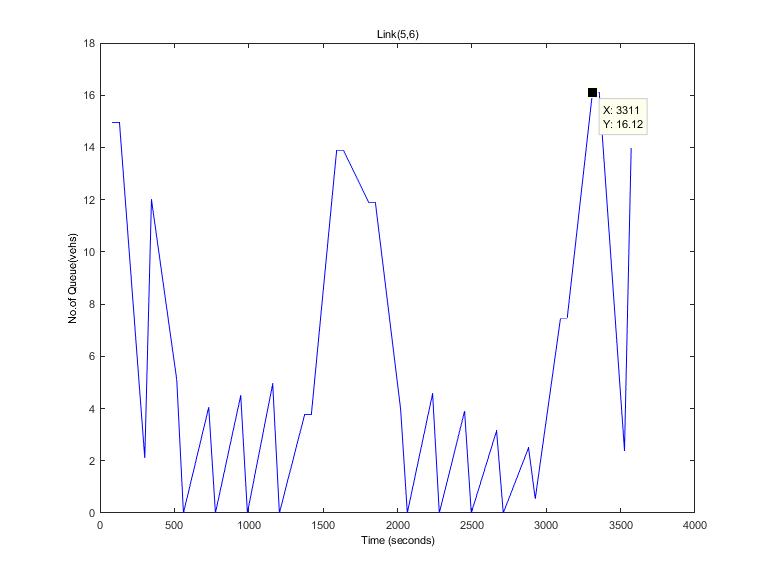

Supplement: S8 File — (JPG) [file pone.0144798.s009.jpg]
